# Supplementary material for: Polycyclic Aromatic Hydrocarbons in Soil at Different Depths under a Long-Term Experiment Depending on Fertilization
Source: Int J Environ Res Public Health. 2022 Aug 22;19(16):10460. doi: 10.3390/ijerph191610460 (PMC9408199; doi:10.3390/ijerph191610460)
Supplement: Supplementary file 1 [file ijerph-19-10460-s001.zip › ijerph-1881415-supplementary.pdf]

Supplementary Materials

Table S1. Design of the field trial.

Series with FYM			Series without FYM		
**Block (repeat)**	**Plot number**	**Mineral fertilisation (SM – Tab. 2)**	**Block (repeat)**	**Plot number**	**Mineral fertilisation (SM – Tab. 2)**
I	1	3	I	48	4
	2	7		47	6
	3	4		46	3
	4	1		45	5
	5	8		44	1
	6	2		43	2
	7	6		42	8
	8	5		41	7
II	9	4	II	40	5
	10	6		39	8
	11	7		38	6
	12	1		37	3
	13	8		36	7
	14	2		35	2
	15	3		34	4
	16	5		33	1
III	17	1	III	32	8
	18	7		31	5
	19	8		30	7
	20	2		29	4
	21	4		28	1
	22	6		27	5
	23	3		26	2
	24	5		25	6

Table S2. Mineral fertilisation regime.

		Sugar beet	*Spring barley*	**Maize**	Spring wheat*									
**No**	**Variant**	**N**				**P**		**K**				**Mg**		
		**Dose [kg ha^{-1}]**												
**1**	**$N_0P_0K_0$**	0	*0*	**0**	0*	**0***	*0*	0	0	**0**	0*	0	*0**	**0**
**2**	**$N_1P_1K_1$**	60	*30*	**60**	40*	**34.9***	*26.2*	66.4	*33.2*	**49.8**	24.9*	0	*0**	**0**
**3**	**$N_2P_1K_1$**	120	*60*	**120**	80*	**34.9***	*26.2*	66.4	*33.2*	**49.8**	24.9*	0	*0**	**0**
**4**	**$N_3P_1K_1$**	180	*90*	**180**	120*	**34.9***	*26.2*	66.4	*33.2*	**49.8**	24.9*	0	*0**	**0**
**5**	**$N_2P_1K_2$**	120	*60*	**120**	80*	**34.9***	*26.2*	132.8	*66.4*	**99.7**	49.8*	0	*0**	**0**
**6**	**$N_2P_1K_3$**	120	*60*	**120**	80*	**34.9***	*26.2*	199.3	*99.7*	**149.7**	74.7*	0	*0**	**0**
**7**	**$N_2P_1K_2Mg$**	120	*60*	**120**	80*	**34.9***	*26.2*	132.8	*66.4*	**99.7**	49.8*	48.2	*18.1**	**24.1**
**8**	**$N_2P_1K_2MgCa$**	120	*60*	**120**	80*	**34.9***	*26.2*	132.8	*66.4*	**99.7**	49.8*	48.2	*18.1**	**24.1**

Table S3. Mean and standard deviation of PAHs 16 content in μg kg^{-1} depending on soil horizon 0-30cm and 30-60 cm in long-term fertilizer experiment

PAHs	Soil horizons			
	0-30 cm		30-60 cm	
	mean	±SD	men	±SD
Naphtalene	0.53	±0.60	0.63	±1.24
Acenaphthylene	3.95	±8.59	1.00	±1.58
Acenaphthene	1.70	±4.72	0.46	±0.55
Fluorene	0.20	±0.49	0.16	±0.09
Phenanthrene	15.55	±19.91	4.75	±6.74
Anthracene	1.42	±2.88	0.82	±1.44
Fluoranthene	12.72	±14.44	28.16	±26.87
Pyrene	9.77	±16.65	17.40	±19.89
Benz(a)anthracene	16.62	±24.22	7.98	±14.81
Chrysene	7.63	±7.55	14.8	±21.18
Benzo(b)fluoranthene	37.61	±34.62	13.20	±16.80
Benz(k)fluoranthene	7.14	±13.30	11.23	±16.15
Benzo(a)pyrene	23.78	±29.21	0.73	±1.35
Ideno(1,2,3-cd)pyrene	8.81	±7.31	2.28	±4.23
Dibenzo(a,h)anthracene	29.63	±25.54	19.77	±24.11
Benzo(g,h,i)perylene	20.97	±27.01	4.65	±9.04
**Sum of 16 PAHs**	**198.04**	**±103.43**	**128.02**	**±89.60**
Sum of 2-rings	0.53	±0.60	0.63	±1.24
Sum of 3-rings	4.56	±5.56	1.44	±1.46
Sum of 4-rings	10.04	±10.38	20.12	±19.10
Sum of 5-rings	22.95	±14.49	10.58	±8.83
Sum of 6-rings	14.90	±15.02	3.46	±5.37
